# Invasion-related circular RNA circFNDC3B inhibits bladder cancer progression through the miR-1178-3p/G3BP2/SRC/FAK axis

**DOI:** 10.1186/s12943-018-0908-8

**Published:** 2018-11-20

**Authors:** Hongwei Liu, Junming Bi, Wei Dong, Meihua Yang, Juanyi Shi, Ning Jiang, Tianxin Lin, Jian Huang

**Affiliations:** Department of Urology and Guangdong Provincial Key Laboratory of Malignant Tumor Epigenetics and Gene Regulation, Sun Yat-sen Memorial Hospital, Sun Yat-sen University, 107th Yanjiangxi Road, Yuexiu District, Guangzhou, 510120 China

**Keywords:** circFNDC3B, miR-1178-3p, G3BP2, SRC/FAK, Bladder cancer

## Abstract

**Background:**

Increasing evidence has revealed that circular RNAs (circRNAs) play crucial roles in cancer biology. However, the role and underlying regulatory mechanisms of circFNDC3B in bladder cancer (BC) remain unknown.

**Methods:**

A cell invasion model was established by repeated transwell assays, and invasion-related circRNAs in BC were identified through an invasion model. The expression of circFNDC3B was detected in 82 BC tissues and cell lines by quantitative real-time PCR. Functional assays were performed to evaluate the effects of circFNDC3B on proliferation, migration and invasion in vitro-, and on tumorigenesis and metastasis in vivo. The relationship between circFNDC3B and miR-1178-3p was confirmed by fluorescence in situ hybridization, pull-down assay and luciferase reporter assay.

**Results:**

In the present study, we identified a novel circRNA (circFNDC3B) through our established BC cell invasion model. We found that circFNDC3B was dramatically downregulated in BC tissues and correlated with pathological T stage, grade, lymphatic invasion and patients’ overall survival rate. Functionally, overexpression of circFNDC3B significantly inhibited proliferation, migration and invasion both in vitro and in vivo. Mechanistically, circFNDC3B could directly bind to miR-1178-3p, which targeted the 5′UTR of the oncogene G3BP2. Moreover, circFNDC3B acted as a miR-1178-3p sponge to suppress G3BP2, thereby inhibiting the downstream SRC/FAK signaling pathway.

**Conclusions:**

CircFNDC3B may serve as a novel tumor suppressive factor and potential target for new therapies in human BC.

**Electronic supplementary material:**

The online version of this article (10.1186/s12943-018-0908-8) contains supplementary material, which is available to authorized users.

## Background

Bladder cancer (BC) is the ninth most common cancer and is among the most frequent types of urinary malignancies worldwide [1]. In 2012, 429,793 patients were diagnosed with BC, and 165, 084 deaths occurred globally [2]. Approximately 25% of newly diagnosed patients present with muscle-invasive BC (MIBC) [3] or metastatic disease [4]. Lymph node (LN) metastasis is a crucial and powerful prognostic factor in BC [5]. Therefore, a profound understanding of the detailed mechanisms underlying LN metastasis in BC is essential for improving the treatment strategies for BC.

Tumor progression is a complex, multistage process. Many oncogenes or pathways have been reported to be involved in cell invasion processes. G3BP2, a member of the Ras-GTPase-activating protein (RasGAP) SH3 domain-binding protein (G3BP) family, is remarkably overexpressed in various human cancers [6, 7] and contributes to tumor invasion [8]. Focal adhesion kinase (FAK) has been confirmed to play a vital role in tumor invasion and metastasis by binding to steroid receptor coactivator (SRC) [9]. Knockdown of G3BP2 limited the migratory and invasive abilities of human lung cancer cells by inhibiting the activity of SRC and FAK [8]. However, the upstream regulatory mechanism of G3BP2 remains elusive.

Circular RNAs (circRNAs) have been identified as a kind of endogenous noncoding RNA [10] and are characterized by covalently closed loop structures without 5′ caps or 3′ polyadenylated tails [11]. In recent years, large amounts of circRNAs have been successfully discovered and identified in different types of cell lines and species by high-throughput sequencing analysis [12]. Many circRNAs have been proven to be dysregulated in various human cancers [13, 14] and have been confirmed to regulate gene expression by sponging miRNAs [13–15]. Although several circRNAs have been reported in BC [16, 17], few tumor invasion-related circRNAs and their involved signaling pathways have been elucidated.

In this study, we identified a novel invasion-related circRNA, circFNDC3B, from published RNA-Seq data of human BC tissues and normal bladder tissues [13] through our established cell invasion model. We further demonstrated that circFNDC3B, which originated from exons 5 and 6 of the FNDC3B gene, was markedly downregulated in BC tissues and cell lines. Low expression of circFNDC3B was significantly associated with pathological stage, grade, lymphatic metastasis and poor survival. Subsequently, we discovered that overexpression of circFNDC3B dramatically inhibited the proliferation, migration and invasion of BC cells via sponging miR-1178-3p to suppress the expression of G3BP2 and inhibit the phosphorylation of SRC/FAK.

## Methods

### Ethics statement and tissue collection

Fresh BC tissues were obtained from patients who were diagnosed with bladder urothelial cancer at Sun Yat-sen Memorial Hospital, Sun Yat-sen University between 2010 and 2016. Eighty-two cases of fresh BC tissues and fifty-six paired adjacent noncancerous tissues (≥3 cm away from the tumor) were immediately frozen in liquid nitrogen and stored at − 80 °C until further use. The experiments were conducted in accordance with the Declaration of Helsinki and approved by the Ethics Committee of Sun Yat-sen Memorial Hospital, Sun Yat-sen University. Written informed consent was obtained from all patients before participation in this study.

### Cell culture

The human invasive BC cell lines T24 and UM-UC-3, and the human immortalized uroepithelium cell line SV-HUC-1 were purchased from ATCC. T24 cells were cultured in RPMI 1640 medium (Gibco, USA); UM-UC-3 cells were cultured in DMEM, and SV-HUC-1 cells were cultured in F-12 K medium (Gibco, USA) supplemented with 10% fetal bovine serum (BI, Israel); and 1% penicillin/streptomycin (Gibco, USA) in a humidified atmosphere of 5% CO_2_ at 37 °C.

### Isolation of invasive and noninvasive BC cell sublines

As previously reported [18], six-well polycarbonate transwell membranes with 8-mm pore inserts (Corning, USA) were used to isolate BC cell T24 sublines. First, T24 cells were serum-starved for 24 h. Then, 1 mL of cell suspension (5 × 10^5^ cells/ml) with serum-free RPMI 1640 medium was seeded into the upper chamber, which was coated with 200 mg/mL of Matrigel (BD Biosciences, USA), and 2.5 mL of RPMI-1640 medium supplemented with 20% FBS was placed into the lower well. The highly invasive cells on the bottom of the membrane and the poorly invasive cells on the top of the membrane were harvested aseptically after incubation for 24 h. Next, the harvested cells were cultured and selected for ten rounds. The cell subline that failed to invade through the membranes in all selection rounds was designated as poorly invasive T24 and the subline that succeeded migrating through the membranes was designated as highly invasive T24. The invasive abilities of the two sublines were then confirmed again by wound healing assay and transwell Matrigel invasion assay.

### Actinomycin D assay

T24 and UM-UC-3 cells were seeded at 1 × 10^5^ cells per well in a 6-well plate overnight and then exposed to 2 mg/L actinomycin D (Sigma, USA) for 4, 8, 12 and 24 h [13]. The cells were harvested at the indicated time points and the stability of circFNDC3B mRNA was analyzed using qRT-PCR. Three independent experiments were performed in triplicate.

### Fluorescence in situ hybridization

When T24 cells seeded in confocal dishes were cultured to 80–90% confluence, the cells were fixed, prehybridized, and hybridized in hybridization buffer [13] with a Cy3-labeled circFNDC3B probe (GenePharma, China) at 37 °C overnight. For the double FISH assay, the Cy3-labeled circFNDC3B probe and Cy5-labeled miR-1178-3p probe were used for hybridization. The signals of the probe were detected by a Fluorescent In Situ Hybridization Kit (GenePharma, China) according to the manufacturer’s protocol. Nuclei were counterstained with Hoechst 33342. The images were captured on ZEISS LSM800 confocal microscope (Carl Zeiss AG, Germany).

### CircRNA plasmid construction and stable transfection

To construct circFNDC3B-overexpressing plasmids, human circFNDC3B cDNA was synthesized and cloned into the plenti-ciR-GFP-T2A vector (IGE Biotech Co, China). Plasmids were transfected into 293 T cells to package lentivirus using X-treme (Sigma, USA) according to the manufacturer’s instructions. T24 and UM-UC-3 cells were infected with the packaged lentivirus and selected with 2 μg/ml puromycin for 3 days. Surviving cells were then used to confirm overexpression efficiency.

### Oligonucleotide transfection

T24 and UM-UC-3 cells were seeded in 6-well plates and cultured to 60–70% confluence before transfection. siRNAs, miRNA mimics, or inhibitors (GenePharma, China) were transiently transfected using Lipofectamine RNAiMax (Invitrogen, USA) according to the manufacturer’s protocol.

### RNase R treatment

RNase R (Epicentre Technologies, USA) was used to degrade linear RNA. Briefly, RNAs extracted from T24 and UM-UC-3 cells were split to two aliquots: one for RNase R digestion and another for control with digestion buffer only [19]. For RNase R digestion, 2 μg of total RNA was mixed with 0.6 μl 10 × RNase R Reaction Buffer and 0.2 μl RNase R (20 U/μl); for control, 2 μg of total RNA was mixed with 0.6 μl 10 × RNase R Reaction Buffer and 0.2 μl DEPC-treated water. Then, the samples were incubated at 37 °C for 30 min [13]. GAPDH in the control group was used as an internal control [19]. Three independent experiments were performed in triplicate.

### RNA preparation and qRT-PCR

RNA isolation of nuclear and cytoplasmic fractions was performed with NE-PER Nuclear and Cytoplasmic Extraction Reagents (Thermo Scientific, USA) according to the manufacturer’s protocol. Total RNA from tissues and cells was extracted with RNAiso Plus (TaKaRa, Japan). Reverse transcription was performed with Prime Script RT Master Mix (TaKaRa, Japan) for circular RNA and mRNA. For miRNA, cDNA was synthesized using a miRNA First Strand cDNA Synthesis Kit (Sangon Biotech, China). Subsequently, the cDNA was subjected to real-time PCR on a Quantstudio™ DX system (Applied Biosystems, Singapore). GAPDH was used as an endogenous control for circRNA and mRNA. The expression of miRNA was normalized to small nuclear U6B (RNU6B). The 2^-∆∆CT^ method was used to calculate relative expression.

### Cell proliferation assay

For the cell viability assay, T24 and UM-UC-3 cells transfected with si-circFNDC3B- or circFNDC3B-overexpression vector were reseeded into 96-well plates (1 × 10^3^ cells per well), and cell viability was assessed by MTS assay (Promega, USA). The absorbance of each well was read at a wavelength of 492 nm on a SPARK 10 M spectrophotometer (Tecan, Austria). For cell colony formation ability, the treated T24 and UM-UC-3 cells were seeded into 6-well plates (500 cells per well). After incubation at 37 °C in a 5% humidified atmosphere for 10 days, colonies were fixed in 4% paraformaldehyde, stained with 0.1% crystal violet, counted and photographed. Three independent experiments were performed in triplicate.

### Cell migration and invasion assays

For wound healing assays, T24 and UM-UC-3 cells were seeded in 6-well plates, and a straight scratch was made using 200 μL pipette tips. Images of wounds were captured at indicated time at three different positions using 10 high-power fields after scratching with a sterile 200 μL pipette. Three independent experiments were performed in triplicate.

For transwell migration and invasion assays, a 24-well transwell chamber (Costar, USA) with or without precoated Matrigel was used to detect cell migratory or invasive abilities according to the manufacturer’s protocol. Cells suspended in 0.2 ml serum-free medium (8 × 10^4^ cells/well for migration, 1 × 10^5^/well for invasion) were added to the upper chambers, and medium supplemented with 10% FBS was applied to the lower chambers. After incubating the cells for 11 h (for T24) and 24 h (for UM-UC-3) at 37 °C; and 5% CO_2_, the cells that migrated to the lower membrane surface were fixed with 4% paraformaldehyde and stained with 1% crystal violet in PBS. The migrated and invaded cells were counted in three randomly selected fields. Three independent experiments were performed in triplicate.

### Biotin-labeled probe pull-down assay

Pull-down assay was performed as previously described [16, 17]. First, to prepare probe-coated beads, the biotinylated circFNDC3B probe or oligo probe (GenePharma, China) was incubated with Streptavidin-Dyna beads M-280 (Invitrogen, USA) at room temperature for 2 h. Then, appoximately 1 × 10^7^ stably overexpressing circFNDC3B or control BC cells were fixed with 1% formaldehyde, lysed in lysis buffer and incubated with probe-coated beads at 4 °C overnight. The probe-Dyna bead-circRNA mixture was washed, followed by incubation with 200 μl lysis buffer and proteinase K at room temperature for 2 h to reverse the formaldehyde crosslinking. Then, the RNA complexes were extracted with RNAiso Plus (TaKaRa, Japan) and detected by qRT-PCR. The results are presented as the means ± SEM from three independent experiments.

### Biotin-labeled miRNA capture

Stably overexpressing circFNDC3B BC cells were transfected with biotin-labeled miRNA mimics or nonsense control (GenePharma, China) for 48 h. Streptavidin-Dyna beads M-280 were washed with lysis buffer; and blocked with yeast tRNA on a rotator at 4 °C for 2 h. The cells were harvested, lysed and incubated with the blocked beads at 4 °C overnight. The abundance of circFNDC3B in bound fractions was tested by qRT-PCR and agarose gel electrophoresis. Three independent experiments were performed in triplicate.

### Dual luciferase reporter assay

Cells were seeded into 24-well plates with 3 × 10^4^ cells per well. After transient transfection with constructed luciferase plasmids and miRNA mimics for 48 h, Rluc activity was measured with a dual-luciferase reporter assay system (Promega, USA) according to the manufacturer′s protocol. Renilla luciferase activity was normalized to the luminescence of firefly luciferase. Three independent experiments were performed in triplicate.

### Western blot analysis

Cells were lysed in RIPA buffer (CWBIO, China) with protease and phosphatase inhibitors (CWBIO, China). Identical quantities of proteins were electrophoresed by SDS-PAGE, transferred onto PVDF membranes and incubated with primary antibodies specific for G3BP2 (1:1000 dilution, Proteintech, USA), SRC (1:800 dilution, ABclonal, China), p-SRC (1:1000 dilution, ABclonal, China), FAK (1:800 dilution, ABclonal, China), p-FAK (1:1000 dilution, ABclonal, China), β-actin (1:1000 dilution, Proteintech, USA), GAPDH (1:1000 dilution, ABclonal, China) at 4 °C overnight, followed by incubation with appropriate HRP-conjugated secondary antibodies at room temperature for 1 h. Signals were detected by Immobilon ECL substrate (Millipore, Germany), and the images were acquired using an Optimax X-ray Film Processor (Protec, Germany).

### HE and immunohistochemistry

Immunohistochemical staining was performed according to published methods [5]. First, 5-μm paraffin sections of tissue samples were stained with HE and immunohistochemistry. The primary antibodies specific for G3BP2 (Proteintech, USA), SRC (ABclonal, China), p-SRC (ABclonal, China), FAK (ABclonal, China), and p-FAK (ABclonal, China) were used at a 1:100 dilution in the experiments. Images were captured using a Nikon Eclipse 80i system with NIS-Elements software (Nikon, Japan).

### Animal experiments

All animal care and experiments were performed according to the guidelines of the National Institutes of Health; and were approved by the Animal Ethics Committee of Sun Yat-sen University. To study the effect of circFNDC3B on tumor growth, circFNDC3B-overexpressing or control UM-UC-3 cells were subcutaneously injected into the upper back of 4-week-old female BALB/c nude mice (5 × 10^6^ cells per mouse). Tumors were measured with a caliper every week. The tumor volume was calculated as (length× width^2^)/2. One month later, the mice were executed, and the excised tumor tissues were further used for evaluation of tumor weight and pathologic examination. To explore the effect of circFNDC3B on lymphatic metastasis, lentivirus-transduced UM-UC-3 cells that stably expressed firefly luciferase were first constructed; subsequently, approximately 5 × 10^5^ of these cells were inoculated into the footpads [5]. After 4 weeks, the popliteal LNs were captured using an in vivo bioluminescence imaging system, then enucleated, weighed and embedded in paraffin.

### Sequences used in this study

The sequences of the primers, oligonucleotides and probes used in this study are listed in Additional file 1: Table S1.

### Statistical analysis

Statistical analyses were carried out using GraphPad Prism 7.0. Chi-square test was performed to analyze the relationship between circFNDC3B levels and clinicopathological characteristics. Kaplan-Meier method and log-rank test were used to calculate overall survival rates. Two-tailed Student’s *t*-test, Wilcoxon rank-sum test, or Mann-Whitney *U*-test were used to determine statistically significant differences between two groups, as appropriate. The Data are presented as the mean ± standard error of the mean (SEM). *P* < 0.05 was considered statistically significant.

## Results

### Identification and characteristics of circFNDC3B in BC cells

**Fig. 1:**
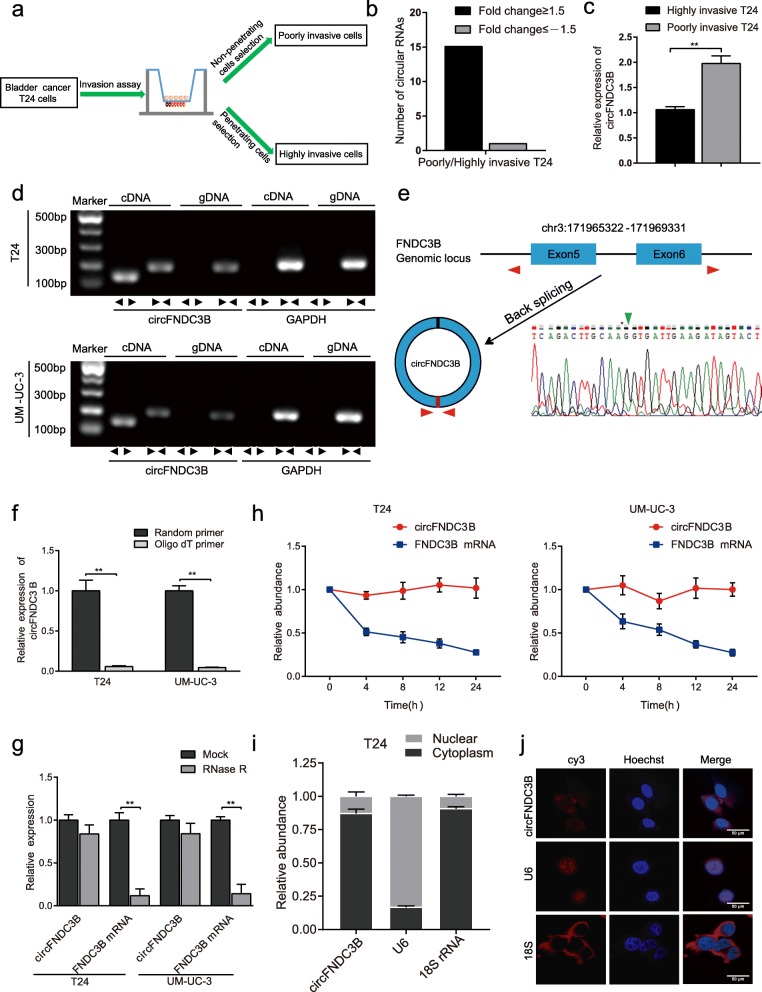
The identification and characteristics of circFNDC3B in BC cells. **a** The general scheme of the establishment of invasive and noninvasive cell sublines from the human BC T24 cell line. **b** Number of differentially changed circular RNAs identified via the T24 cell invasion model. **c** Relative expression of circFNDC3B in poorly and highly invasive T24 cell sublines. **d** The PCR products of circFNDC3B and linear FNDC3B were tested by gel electrophoresis. Divergent primers amplified circFNDC3B in cDNA but not genomic DNA (gDNA). Convergent primers amplified linear FNDC3B in both cDNA and gDNA. GAPDH was used as a linear control. **e** The formation of circFNDC3B. circFNDC3B derived from back-spliced exons 5 and 6 of genomic FNDC3B. The existence of circFNDC3B was confirmed by Sanger sequencing. The green arrow represents the back-splice junction of circFNDC3B. **f q**RT-PCR analysis of circFNDC3B using random primers and oligo-dT primers,respectively in reverse transcription experiments. CircFNDC3B is absent in polyA-enriched samples. **g** The expression of circFNDC3B and FNDC3B mRNA was measured by qRT-PCR in T24 and UM-UC-3 cells treated with or without RNase R. **h** qRT-PCR analysis of circFNDC3B and FNCDC3B mRNA in T24 and UM-UC-3 cells treated with actinomycin D at the indicated time points. **i** and **j** CircFNDC3B was mainly located in the cytoplasm, as confirmed by the nuclear mass separation assay and FISH, in T24 cells. Nuclei were stained with Hoechst33342. Scale bar, 50 μm. Data are shown as the means±SEM of three experiments. **P < 0.01 (Student’s *t*-test)

**Fig. 2:**
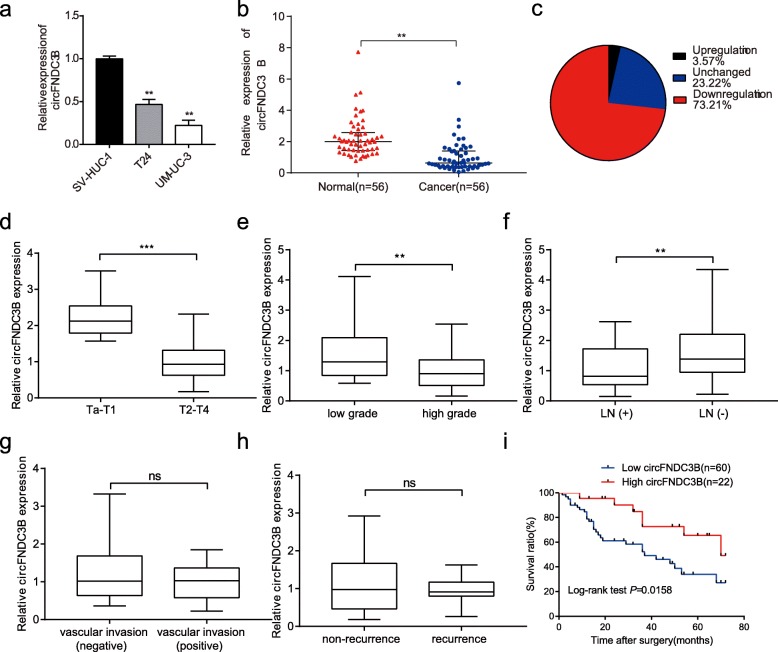
The expression and clinical significance of circFNDC3B in BC. **a** The expression of circFNDC3B in BC cell lines was detected by qRT-PCR. **b** and **c** qRT-PCR analysis of circFNDC3B in 56 paired BC and adjacent noncancerous tissues. **d-h** The expression levels of circFNDC3B in BC patients with different pathological T stages, grades, lymph node status, and vascular invasion status. LN represents lymph node. **i** Kaplan-Meier survival curve indicated that patients with low circFNDC3B expression had low survival rates. a, **P < 0.01 (Student’s *t*-test); b, **P < 0.01 (Wilcoxon rank-sum test); d-h, **P < 0.01, ***P < 0.001 (Mann-Whitney *U*-test)

We first analyzed and selected 62 differentially expressed circRNAs (fold change≥2.0 and *P* < 0.05) from RNA-Seq data of BC tissues and normal tissues [13]. Twenty-six circRNAs were detected in BC cell lines using qRT-PCR (data not shown). To select invasion-related circRNAs, we tested expression of the 26 circRNAs via the established cell invasion model (Fig. 1a) by qRT-PCR. Sixteen differentially changed circRNAs (fold change≥1.5) were screened from highly and poorly invasive T24 cells, among which 15 circRNAs may inhibit migration and invasion in BC, and only one circRNA may promote invasion in BC (Fig. 1b). Then, we focused on circFNDC3B (hsa_circ_0006156), which expressed at low levels in both highly invasive T24 cells and BC tissues, for further study (Fig. 1c, 2b).

CircRNAs share the same sequences with their linear counterparts except for the back-splice junction area, so the circular form of FNDC3B should be further verified. We first designed two sets of primers for FNDC3B: convergent primers that were expected to amplify only the linear form, FNDC3B mRNA, and divergent primers to amplify only the circular form, circFNDC3B. cDNA and genomic DNA (gDNA) were used as templates, and the specificity of the qRT-PCR was validated by 1% agarose gel electrophoresis. As expected, the single and distinct product of the expected size was amplified using the divergent primers in only cDNA, while no product was amplified with the divergent primers in gDNA (Fig. 1d). Sanger sequencing of the PCR product was further performed to confirm the head-to-tail splicing. The results showed that the sequence of the amplified product was consistent with that of circFNDC3B in circBase, which indicated that circFNDC3B was derived from exon 5 and exon 6 of the FNDC3B gene (Fig. 1e). Furthermore, circFNDC3B expression was significantly lower or undetectable when oligo-dT primers were used in the reverse transcription experiments (Fig. 1f), indicating that circFNDC3B was depleted in poly (A)-enriched samples. Moreover, when T24 and UM-UC-3 were treated with RNase R, a highly processive 3′ to 5′ exoribonuclease, we found that circFNDC3B was resistant to digestion induced by RNase R (Fig. 1g). We next studied the stability of circFNDC3B in T24 and UM-UC-3 cells. Total RNA was harvested at the indicated time points after treatment with actinomycin D, an inhibitor of transcription. As a result, the transcript half-life of circFNDC3B exceeded 24 h, whereas the transcript half-life of linear FNDC3B was approximately 4 h in both T24 and UM-UC-3 cells (Fig. 1h), indicating that circFNDC3B is more highly stable than linear FNDC3B in BC cells. In addition, circFNDC3B was predominantly located in the cytoplasm, as confirmed by the nuclear mass separation assay and fluorescence in situ hybridization (FISH) (Fig. 1i-j). Taken together, these results indicate that circFNDC3B is a highly stable and cytoplasmic circRNA derived from exons 5 and 6 of the FNDC3B gene locus.

### CircFNDC3B is expressed at low levels in BC and correlates with progression and prognosis

First, we examined the expression of circFNDC3B in different BC cell lines by qRT-PCR. As shown in Fig. 2a, circFNDC3B expression was downregulated in BC cell lines normalized to SV-HUC-1 cells. Next, we investigated circFNDC3B expression in 56 pairs of BC tissues and adjacent noncancerous tissues. qRT-PCR analysis showed that circFNDC3B was expressed at low levels in 73.2% of cancerous tissues than in adjacent noncancerous tissues (Fig. 2b-c). Then, the expression of circFNDC3B was analyzed in a total of 82 patients. Patients with a high grade, a highly pathological T stage, and lymphatic metastasis had lower circFNDC3B expression (Fig. 2d-f). However, no statistical difference of the circFNDC3B expression levels was observed in the vascular invasion or recurrence group, compared to their corresponding non-vascular invasion or non-recurrence group, respectively (Fig 2g-h). To further study the correlation between circFNDC3B expression and clinicopathological characteristics, tumors were classified into low and high circFNDC3B expression groups. Chi-square test was then conducted to assess clinicopathological factors between the two groups. As shown in Table 1, low circFNDC3B expression was positively correlated with grade, pathological T stage, and lymphatic metastasis. However, the circFNDC3B expression levels harbored no correlation with other clinical parameters, such as age, gender, tumor size, vascular invasion or recurrence. Furthermore, the Kaplan-Meier analysis illustrated that BC patients with low circFNDC3B expression exhibited poorer survival compared with BC patients with high circFNDC3B expression (Fig. 2i).

**Table 1:** Correlation between circFNDC3B expression and clinical features in bladder cancer

Characteristics		circFNDC3B expression		
	No. (%)	Low (%)	High (%)	*P*-value
Gender				
Male	49 (59.8)	37 (75.5)	12 (24.5)	0.560
Female	33 (40.2)	23 (69.7)	10 (30.3)	
Age				
< 60	29 (35.4)	21 (72.4)	8 (27.6)	0.909
≥ 60	53 (64.6)	39 (73.6)	14 (26.4)	
Tumor size				
< 3 cm	47 (57.3)	37 (78.7)	10 (21.3)	0.189
≥ 3 cm	35 (42.7)	23 (65.7)	12 (34.3)	
Pathology stage				
pTa-pT1	28 (34.1)	13 (46.4)	15 (53.6)	< 0.0001***
pT2-T4	54 (65.9)	47 (87.0)	7 (13.0)	
Grade				
Low	27 (32.9)	15 (55.6)	12 (44.4)	0.012*
High	55 (67.1)	45 (81.8)	10 (18.2)	
Lymphatic metastasis				
Yes	46 (56.1)	38 (82.6)	8 (17.4)	0.029*
No	36 (43.9)	22 (61.1)	14 (38.9)	
Vascular invasion				
Yes	11 (13.4)	8 (72.7)	3 (27.3)	0.975
No	71 (86.6)	52 (73.2)	19 (26.8)	
Recurrence				
Yes	20	14 (70.0)	6 (30.0)	0.713
No	62	46 (74.2)	16 (25.8)	
Total	82	60	22	

Chi-square test. ***P < 0.0001, *P < 0.05

### CircFNDC3B inhibits proliferation, migration and invasion of BC cells in vitro

**Fig. 3:**
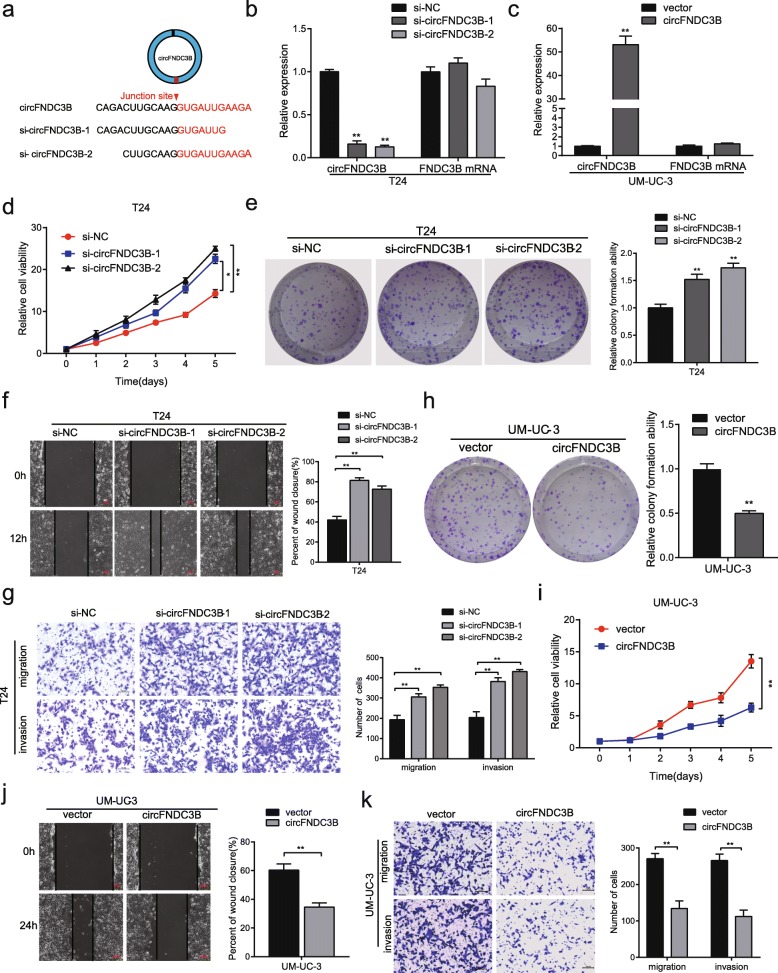
circFNDC3B exerts anti-tumor effects in BC cells. **a** Schematic illustration of two targeted siRNAs. si-circFNDC3B targets the back-splice junction of circFNDC3B. **b** and **c** qRT-PCR analysis of circFNDC3B and FNDC3B mRNA in T24 cells treated with siRNAs (**b**) and UM-UC-3 cells stably overexpressing circFNDC3B (**c**). **d** and **e** The cell proliferative ability was assessed by MTS and colony formation assay after knocking down circFNDC3B in T24 cells. (**f**-**g**) Cell migratory and invasive capabilities were assessed by wound healing assay and transwell assay after knocking down circFNDC3B in T24 cells. (**h**-**i**) The cell proliferative ability was assessed by MTS and colony formation assay after overexpressing circFNDC3B in UM-UC-3 cells. (**j**-**k**) The cell migration and invasion capabilities were assessed by wound healing assay and transwell assay after overexpressing circFNDC3B in UM-UC-3 cells. The red scale bar indicates 200 μm; the black scale bar indicates 100 μm. Data are presented as the means±SEM of three experiments. *P < 0.05, **P < 0.01(Student’s *t*-test)

To test whether circFNDC3B could affect the biological behavior of BC cells, loss-of-function and gain-of-function assays were performed. We first designed two siRNAs targeting the back-splice region of circFNDC3B (Fig. 3a). After transfection of the siRNAs into T24 and UM-UC-3 cells, circFNDC3B was successfully knocked down, and FNDC3B mRNA was not affected (Fig. 3b, Additional file 2: Figure S1a). In addition, a circFNDC3B-overexpressing plasmid was successfully constructed, and no significant change in FNDC3B mRNA was observed (Fig. 3c, Additional file 2: Figure S1b).

MTS assay and colony formation experiments revealed that knock-down of circFNDC3B increased cell growth and colony-forming abilities in both T24 and UM-UC-3 cells compared to control cells (Fig. 3d-e, Additional file 2: Figure S1c-d). Wound healing assays and transwell assays confirmed that migratory and invasive abilities were significantly promoted (Fig. 3f-g, Additional file 2: Figure S2a-b). In contrast, stable overexpression of circFNDC3B showed the reverse effects (Fig. 3h-k, Additional file 2: Figure S1e-f, S2c-d). Taken together, these experiments demonstrated that circFNDC3B inhibits the proliferation, migration and invasion of BC cells in vitro.

### CircFNDC3B directly binds to miR-1178-3p in BC cells

**Fig. 4:**
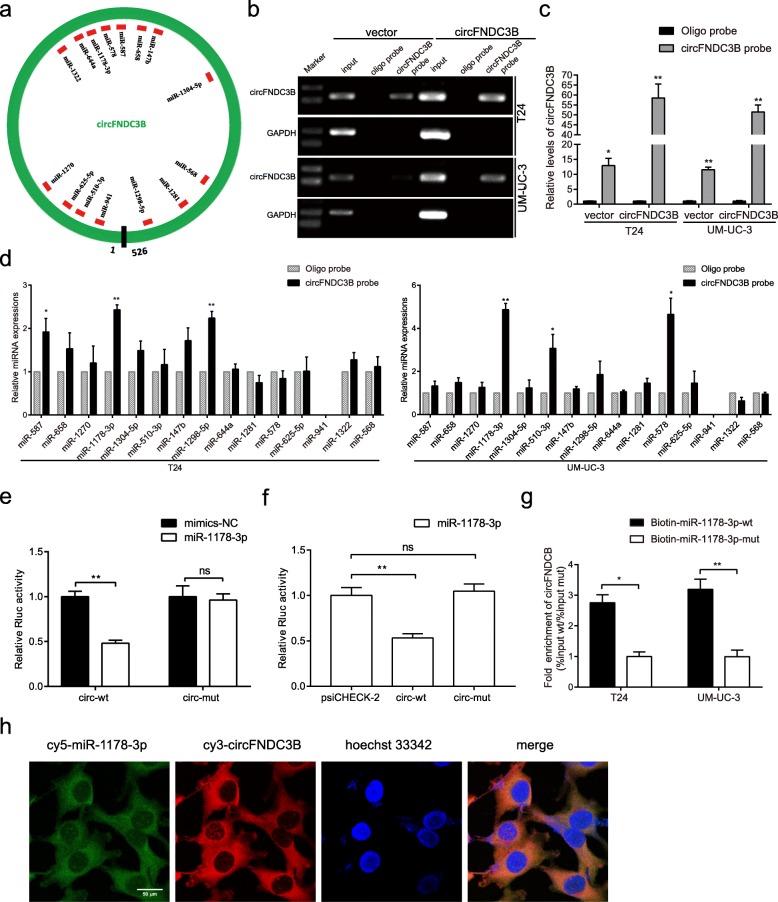
circFNDC3B could serve as a miR-1178-3p sponge in BC cells. **a** Potential target miRNAs of circFNDC3B were predicted by CircInteractome. **b** and **c** RNAs pulled down by a circFNDC3B probe were tested by qRT-PCR (**b**) and gel electrophoresis (**c**). GAPDH was used as a negative control. Relative levels of circFNDC3B were normalized to input. **d** qRT-PCR analysis of miRNA candidates in T24 and UM-UC-3 lysates. **e** The Renilla luciferase activity of wild type or mutant circFNDC3B in the miR-1178-3p mimics or NC group. **f** The Renilla luciferase activity analysis in 293 T cells after transfection with miR-1178-3p mimics+psiCHECK-2 empty vector, miR-1178-3p mimics+wild type circFNDC3B or miR-1178-3p mimics+mutant circFNDC3B. Data are presented as the ratio of Renilla luciferase activity to firefly activity. **g** The RNAs captured by biotinylated wild-type or mutant miR-1178-3p in T24 and UM-UC-3 cells were quantified by qRT-PCR. **h** Colocalization of circFNDC3B and miR-1178-3p was conducted by FISH. Nuclei were stained with Hoechst33342. Scale bar, 50 μm. Data are presented as the means±SEM of three experiments. *P < 0.05, **P < 0.01 (Student’s *t*-test)

CircFNDC3B was predominantly located in the cytoplasm, suggesting that it may function posttranscriptionally. Twenty-one potential miRNAs associated with circFNDC3B were predicted by CircInteractome (Fig. 4a, Additional file 3). By pull-down assay using a biotin-coupled circFNDC3B probe, obvious enrichment of circFNDC3B was pulled down compared with an oligo-negative probe in T24 and UM-UC-3 cells, especially in the circFNDC3B-overexpressing group (Fig. 4b-c). We purified the circFNDC3B bound RNA complex and analyzed the 21 candidate miRNAs by qRT-PCR. We found abundant enrichment of miR-1178-3p pulled down by circFNDC3B in both T24 and UM-UC-3 cells (Fig. 4d). Subsequently, a circFNDC3B fragment with wild-type or mutant complementary binding sites was constructed and inserted downstream of the luciferase reporter gene psiCHECK-2. We first cotransfected a luc-circFNDC3B-wt or a luc-circFNDC3B-mut plasmid, respectively, with miR-1178-3p mimics or NC in HEK-293 T cells. The dual-luciferase reporter assay revealed that the renilla luciferase (Rluc) activity of circFNDC3B-wt but not circFNDC3B-mut obviously reduced in the miR-1178-3p mimics group, compared with their NC group (Fig. 4e). Then, a luc-circFNDC3B-wt or a luc-circFNDC3B-mut plasmid and miR-1178-3p mimics were cotransfected into HEK-293 T cells. Interestingly, Rluc activity of circFNDC3B-wt also significantly decreased, compared with psiCHECK-2 empty vector. However, no significant change in Rluc activity was observed when the corresponding target sites were mutated (Fig. 4f, Additional file 2: Figure S3a). Furthermore, we transfected biotinylated miR-1178-3p mimics into T24 and UM-UC-3 cells stably overexpressing circFNDC3B, and the RNA captured by miR-1178-3p was then tested by qRT-PCR. Consistent with the circRNA-miRNA pull-down assay, biotinylated miR-1178-3p mimics captured more circFNDC3B than biotinylated miR-1178-3p-mut, demonstrating that miR-1178-3p could also directly bind to circFNDC3B (Fig. 4g); Additionally, the double FISH assay confirmed the colocalization of circFNDC3B and miR-1178-3p in the cytoplasm (Fig. 4h). Together, these results indicate that circFNDC3B could directly bind to miR-1178-3p.

### miR-1178-3p exerts an oncogenic role and targets the 5’UTR of G3BP2 in BC

**Fig. 5:**
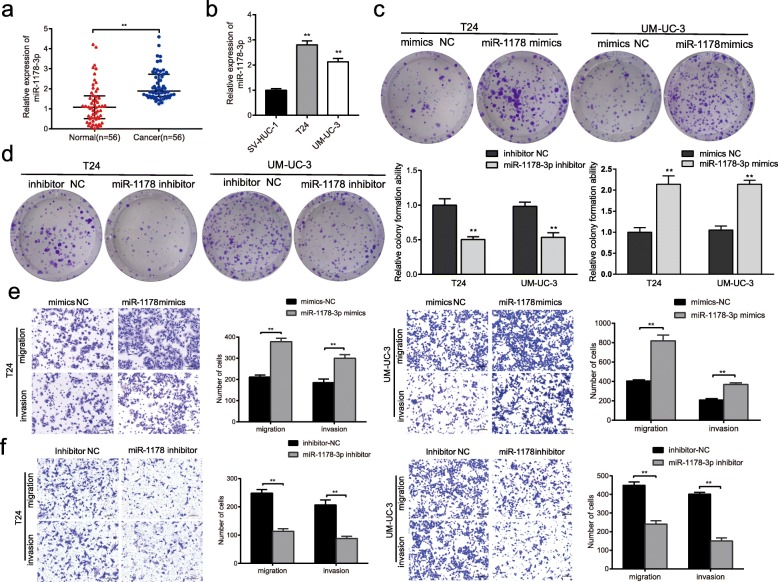
miR-1178-3p is highly expressed in BC tissues and exerts oncogenic roles in BC cells. **a** and **b** Relative expression levels of miR-1178-3p in BC tissues (**a**) and cell lines (**b**) were measured by qRT-PCR. **c** and **d** The colony formation ability of T24 and UM-UC-3 cells transfected with miR-1178-3p mimics or inhibitor was tested by colony formation assay. **e** and **f** The migratory and invasive abilities of T24 and UM-UC-3 cells transfected with miR-1178-3p mimics or inhibitors were measured by transwell migration and Matrigel invasion assays. a, **P < 0.01 (Wilcoxon rank-sum test); b-f, data are presented as the means±SEM of three experiments. *P < 0.05, **P < 0.01 (Student’s *t*-test)

miR-1178 has been reported to act as an oncomiR during pancreatic cancer tumorigenesis [20]. We found that miR-1178 was also upregulated in human BC tissues and cell lines compared with that in normal bladder tissues and SV-HUC-1 cells (Fig. 5a-b). Functionally, proliferative, migratory and invasive abilities were significantly enhanced after T24 and UM-UC-3 cells were treated with miR-1178-3p mimics. In contrast, the opposite results were obtained when the cells were transfected with miR-1178-3p inhibitor (Fig. 5c-f).

**Fig. 6:**
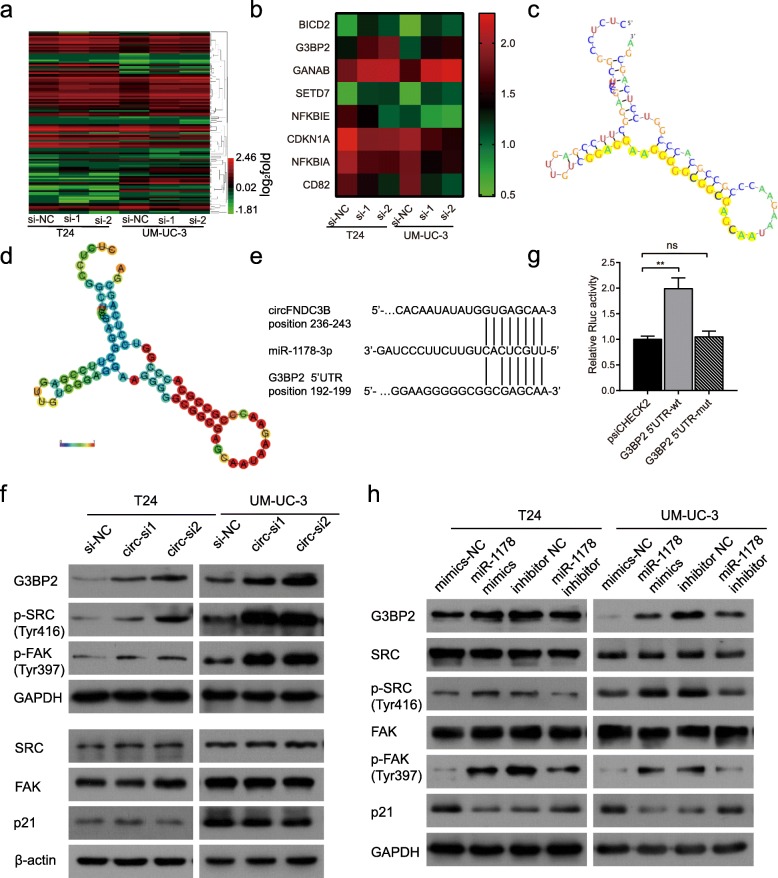
The identification and verification of circFNDC3B-related downstream genes in BC cells. **a** Differentially expressed genes in T24 and UM-UC-3 cells after knockdown of circFNDC3B. **b** Heat map analysis of tumor-related genes identified in this study. Red indicates upregulated expression; green indicates downregulated expression. **c** and **d** The possible binding sites of the 5’UTR of G3BP2 with miR-1178-3p were predicted by RegRNA2.0. **c** Graph of predicted RNA secondary structure. The yellow region indicates the RNAfold predicted structure of the motif. **d** RNAfold reliability information of pair probabilities. Minimum free energy = − 34.02. **e** Sequence alignment of miR-1178-3p with circFNDC3B (predicted by Targetscan) and 5’UTR of G3BP2 (predicted by RegRNA2.0). **f** Immunoblotting of G3BP2, SRC, p-SRC, FAK, p-FAK after knocking down circFNDC3B in T24 and UM-UC-3 cells. **g** Renilla luciferase activity in UM-UC-3 cells cotransfected with miR-1178-3p mimics and wild-type or mutant G3BP2 5’UTR luciferase reporter. Data are presented as the ratio of Renilla luciferase activity to firefly activity. **h** Immunoblotting of G3BP2, SRC, p-SRC, FAK, p-FAK in T24 and UM-UC-3 cells transfected with miR-1178-3p mimics or inhibitor. Data are shown as the means±SEM of three experiments. **P < 0.01 (Student’s *t*-test)

To find the target genes of miR-1178-3p that could be regulated by circFNDC3B, T24 and UM-UC-3 cells transfected with circFNDC3B siRNAs were performed for gene expression profiling. A total of 116 differentially changed genes were screened in both T24 and UM-UC-3 cells treated with circFNDC3B siRNA1 and siRNA2 (Fig. 6a, Additional file 4); We then chose 8 cancer-related genes that were previously reported for further target gene selection (Fig. 6b). qRT-PCR results revealed that 3 genes (p21, NFKBIA and NFKBIE) were significantly downregulated and that 4 genes (G3BP2, GANAB, SETD7, and BICD2) were upregulated after downregulation of circFNDC3B in both T24 and UM-UC-3 cells (Additional file 2: Figure S3b-c). miRNAs have been reported to mainly target the 3’UTR of mRNAs, resulting in the repression of translation or degradation of mRNA targets [21, 22]. However, miRNAs may also target the 5’UTR, generally inducing the activation of translation [23, 24]. We found that miR-1178-3p harbored seed sequence recognition sites that were complementary to the 3’UTR of p21 and 5’UTR of G3BP2, as predicted by Targetscan and RegRNA2.0, while no complementary sites were predicted with other genes (Fig. 6c-e, Additional file 2: Figure S3d).

As a member of the G3BP family, G3BP2 has been to be reported remarkably overexpressed in many human cancers [8, 25, 26], including BC [6]. Western blot showed that miR-1178-3p mimics observably promoted G3BP2 protein expression and suppressed p21 protein expression, whereas miR-1178-3p inhibitor exhibited opposite results (Fig. 6h). However, only G3BP2 was regulated by circFNDC3B (Fig. 6f). To further validate whether miR1178-3p directly targets G3BP2, the 5’ UTR of G3BP2 was constructed and cloned into the psiCHECK-2 vector. We found that Rluc activity was significantly increased after cotransfection of miR-1178-3p mimics with G3BP2–5’ UTR compared with the mutant vector (Fig. 6g, Additional file 2: Figure S3e). These results indicated that miR-1178-3p could bind to the 5’ UTR of G3BP2, resulting in upregulation of G3BP2.

G3BP2 has been reported to promote the phosphorylation of SRC and FAK, which play a vital role in cancer invasion and metastasis [8]. Our results showed that upregulation of miR-1178-3p did not lead to a significant change in SRC expression. However, Tyr416 phosphorylation of SRC remarkably increased (Fig. 6h). As a downstream effector of SRC activation, phosphorylation of FAK at Tyr397 could create a high-affinity binding site for SRC [27]; We next examined FAK expression and its activity by phosphorylation of Tyr397. We observed that FAK activation was also increased in miR-1178-3p mimic-treated cells. The opposite effect was observed when cells were treated with miR-1178-3p inhibitor (Fig. 6h).

### CircFNDC3B downregulates G3BP2 expression and inhibits the phosphorylation of SRC/FAK by sponging miR-1178-3p

**Fig. 7:**
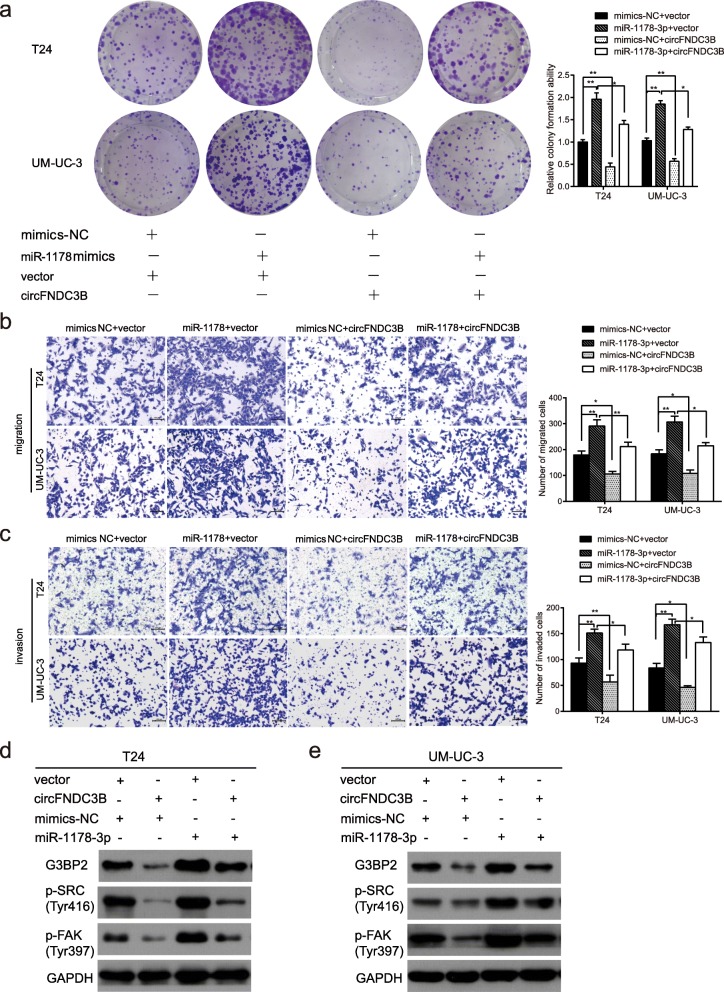
Overexpression of circFNDC3B reverses the oncogenic roles of miR-1178-3p in BC cells. **a**-**c** The colony formation (**a**), migration (**b**), and invasion (**c**) abilities enhanced by miR-1178-3p were reversed after cotransfection with circFNDC3B using colony formation assay, transwell migration assay and Matrigel invasion assay. Scale bar, 100 μm. **d** and **e** The upregulation of G3BP2, p-SRC and p-FAK in T24 and UM-UC-3 cells transfected with miR-1178-3p mimics was reversed by overexpression of circFNDC3B as detected by western blot. Data are presented as the means±SEM of three experiments. *P < 0.05, **P < 0.01 (Student’s *t*-test)

To further verify whether circFNDC3B exerts its inhibitory effect on BC by sponging miR-1178-3p, we performed a “rescue” experiment to examine the functional interaction of circFNDC3B/miR-1178-3p. We found that the proliferative, migratory and invasive abilities of overexpressed circFNDC3B cells transfected with miR-1178-3p mimics obviously decreased compared with those of BC cells transfected with empty vector and miR-1178-3p mimics (Fig. 7a-c), suggesting that circFNDC3B suppresses BC progression and partly eliminates the oncogenic effect of miR-1178-3p. Next, the effects of circFNDC3B on target genes were studied. Western blot analysis showed that the G3BP2 protein level was upregulated after circFNDC3B was knocked down (Fig. 6f), whereas G3BP2 was downregulated after overexpressing circFNDC3B, but this effect could be partially abrogated by ectopic expression of miR-1178-3p (Fig. 7d-e). Furthermore, circFNDC3B-siRNA treatments significantly increased the phosphorylation of SRC and FAK (Fig. 6f), while overexpression of circFNDC3B led to decreased phosphorylation of SRC and FAK, and this effect was also abolished by cotransfection of miR-1178-3p mimics (Fig. 7d-e). These data indicated that circFNDC3B could act as a miR-1178-3p sponge to regulate G3BP2 expression; and an inactive SRC/FAK signaling pathway.

### Silencing of G3BP2 reverses the oncogenic effect induced by knockdown of circFNDC3B

**Fig. 8:**
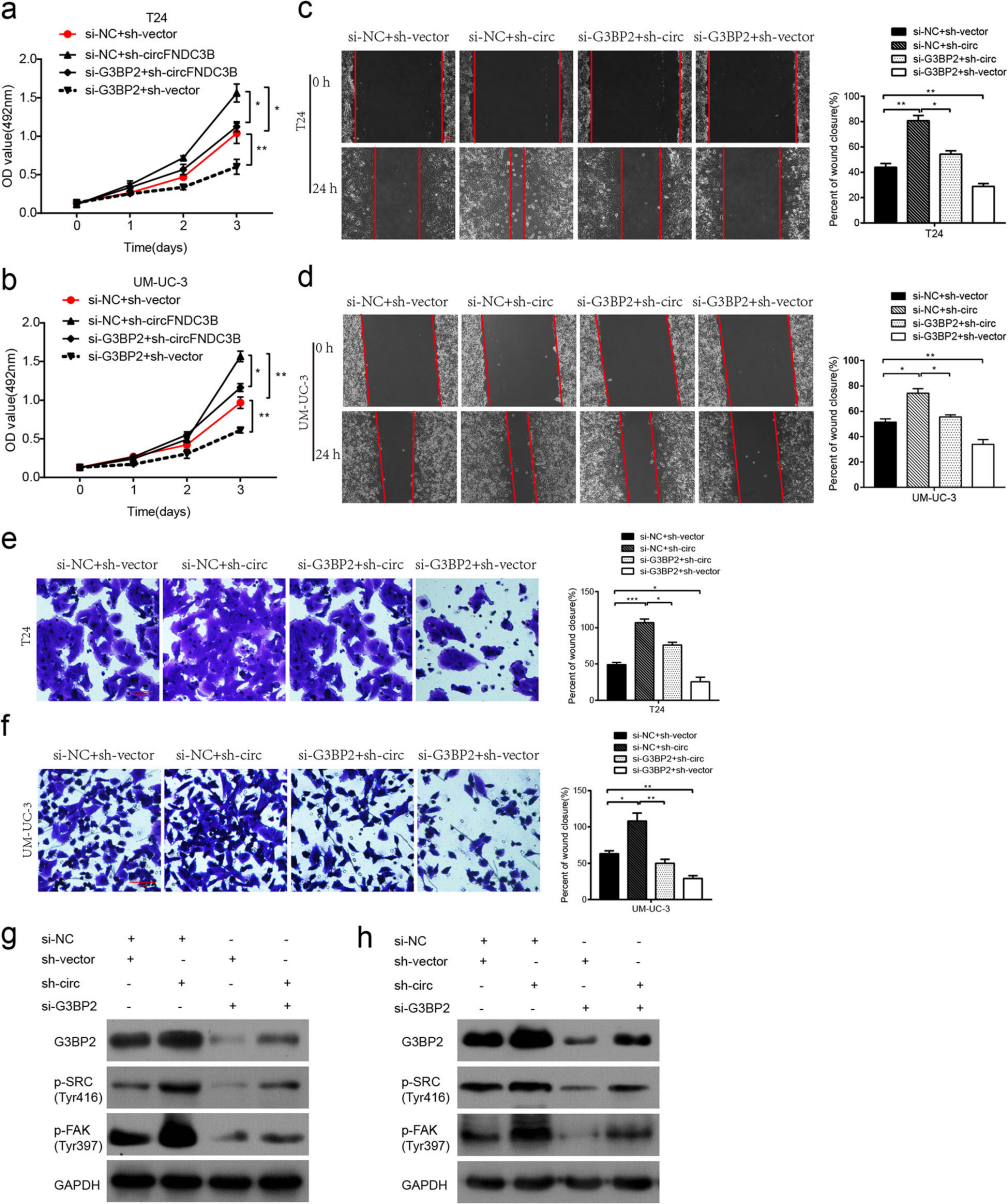
Knockdown of G3BP2 abolishes the oncogenic effects induced by downregulation of circFNDC3B. T24 or UMUC-3 cells were transfected with si-NC + sh-vector, si-NC + sh-circFNDC3B, si-G3BP2 + sh-circFNDC3B or si-G3BP2 + sh-vector. **a** and **b** The cell proliferation was measured by MTS assays. **c** and **d** The migratory ability was assessed by wound healing assays. **e** and **f** The invasive capacity was evaluated by transwell Matrigel invasion assays. **g** and **h** The G3BP2, p-SRC and p-FAK protein levels were detected by western blot assays. Data are presented as the means±SEM of three experiments. *P < 0.05, **P < 0.01, ***P < 0.001 (Student’s *t*-test). Scale bar, 200 μm

To investigate whether manipulation the level of G3BP2 could recreate the effects of circFNDC3B modulation, we transfected G3BP2 siRNA into T24 or UM-UC-3 cells that stably knock down circFNDC3B (sh-circFNDC3B). Subsequent cell proliferation assay demonstrated that sh-circFNDC3B promoted cell viability of T24 cells, while knockdown of G3BP2 reversed this effect (Fig. 8a-b). In addition, wound healing assays demonstrated that cell migratory ability was strengthened by sh-circFNDC3B, but was impaired by transfection with si-G3BP2 (Fig. 8c-d). Analogously, transwell Matrigel invasion assay revealed that cell invasive ability was also enhanced by sh-circFNDC3B, but was abolished by knockdown of G3BP2 (Fig. 8e-f). Moreover, knockdown of G3BP2 also abrogated sh-circFNDC3B-induced increase on G3BP2, p-SRC, or p-FAK expression (Fig. 8g-h). Collectively, these results indicate that G3BP2 plays a vital role in the circFNDC3B/G3BP2/SRC/FAK axis.

### CircFNDC3B suppresses tumor growth and lymphatic metastasis of BC cells in vivo

**Fig. 9:**
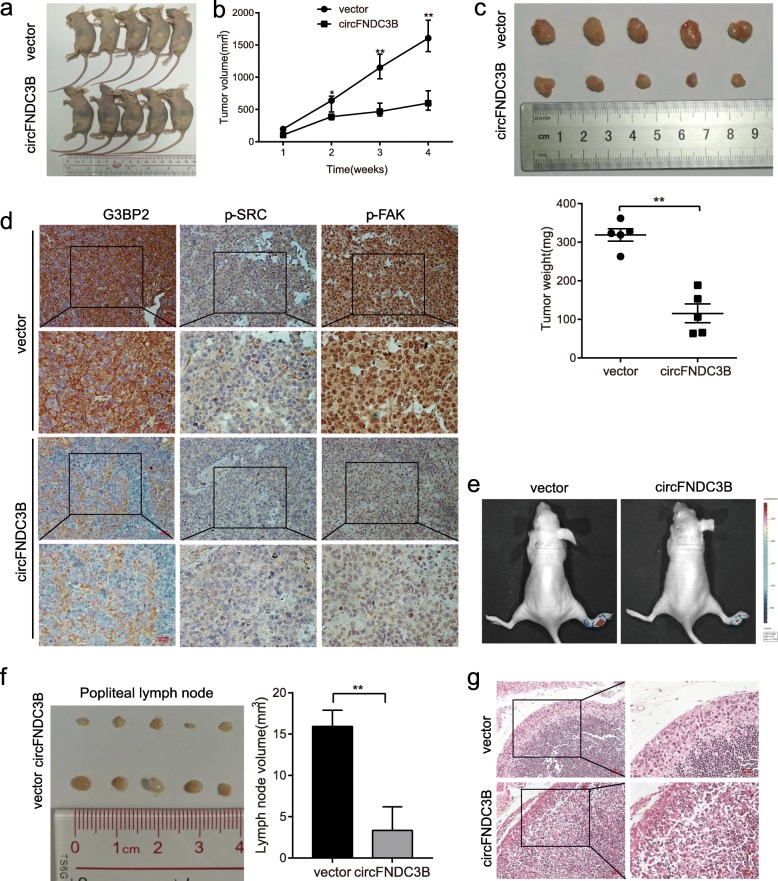
Overexpression of circFNDC3B suppresses the growth and lymphatic metastasis of BC cells in vivo. **a** Image of BALB/c nude mice injected with UM-UC-3 cells subcutaneously (5 × 10^6^ cells per mouse, *n* = 5 for each group). **b** Analysis of tumor volume of mice measured every week. **c** Image of subcutaneous xenograft tumors. Tumor weights were significantly decreased in circFNDC3B–treated mice. **d** IHC of G3BP2, p-SRC, p-FAK in the subcutaneous tumors. Scale bar, 200 μm. **e** Representative image of metastatic popliteal LN captured by an in vivo bioluminescence imaging system. **f** The image of excised popliteal LNs. The LN volume significantly decreased in circFNDC3B-treated mice. **g** HE staining of popliteal LNs. Scale bar, 200 μm. Data indicate the means±SEM of three experiments, *P < 0.05, **P < 0.01 (Student’s *t*-test)

To explore the effects of circFNDC3B in vivo, circFNDC3B-overexpressing UM-UC-3 cells and negative control cells were subcutaneously injected into BALB/c nude mice. The results revealed that the growth rate and tumor weight of tumors in the circFNDC3B-overexpressing group were significantly inhibited (Fig. 9a-c). These subcutaneous tumor tissues were further applied for immunohistochemical staining. The expression levels of G3BP2, p-SRC and p-FAK were significantly inhibited in the circFNDC3B-overexpressing group compared with those in the control group (Fig. 9d). We further injected firefly luciferase expressing UM-UC-3 cells into the footpads of nude mice to explore the effect of circFNDC3B on lymphatic metastasis. As expected, the luminescence of popliteal LNs was weaker or undetectable (Fig. 9e), and the volumes of the popliteal LNs were significantly smaller (Fig. 9f) in the circFNDC3B/mice than in the corresponding control mice, indicating that overexpression of circFNDC3B could inhibit lymphatic metastasis of BC (Fig. 9g).

## Discussion

CircRNAs are a large class of widespread and highly stable endogenous noncoding RNAs that have been identified by high-throughput sequencing and bioinformatics analysis in recent years [15, 28]. An increasing number of circRNAs have been confirmed to be dysregulated in various human cancers [13, 14, 16, 17]. In the present study, we identified a novel circular RNA termed circFNDC3B that was aberrantly downregulated in BC tissues. Low expression of circFNDC3B was correlated with high grade, lymphatic metastasis and poor prognosis.

Invasion and metastasis are the greatest obstacles to successful tumor treatment and are the leading reason for the resultant mortality of patients with BC [29], which urges us to identify invasion-related genes and elucidate the molecular mechanisms resulting in tumor invasion and metastasis. In our study, we constructed an invasion model by isolating highly and poorly invasive cell sublines from T24 using the repeated transwell method, which has been successfully used to investigate tumor metastasis [18]. We successfully identified invasion-related circRNAs from RNA-Seq data of BC tissues through the cell invasion model. CircFNDC3B, one of the most differentially expressed circRNAs between highly and poorly invasive T24 sublines, was further confirmed to inhibit proliferation, migration and invasion in vitro and to suppress tumor growth and lymphatic metastasis in vivo, suggesting its tumor-suppressive effect.

A general phenomenon was discovered: circRNAs were described as pivotal gene regulators in humans mainly due to their posttranscriptional function. CircRNAs contain multiple miRNA-binding sites or miRNA response elements (MREs) that can function as miRNA sponges [15, 28]. For example, circHIPK3 could bind to a host of miRNAs and act as a miRNA sponge in human cancers [13, 16, 30]. hsa_circ_0000977 upregulates oncogene PLK1 by sponging miR-874-3p in pancreatic ductal adenocarcinoma [31]. In our study, circFNDC3B was confirmed to be mainly located in the cytoplasm; and to harbor numerous miRNA-binding sites predicted by CircInteractome, suggesting that circFNDC3B may also function as miRNA sponge in BC. A series of experiments such as biotin-labeled probe pull-down assay, dual- luciferase reporter assay and biotin-labeled miRNA capture assay, confirmed that circFNDC3B directly binds to miR-1178-3p. Subsequent “rescue” experiments confirmed that circFNDC3B could reverse the oncogenic roles of miR-1178-3p, suggesting that circFNDC3B acts as a miR-1178-3p sponge in BC.

Mature miRNAs have been reported to bind to the 3’UTR of target mRNAs, leading to the repression of translation or induction of degradation of target mRNAs [21]. To date, most studies have revealed that circRNAs positively regulate target genes of miRNAs. For example, circular RNA_LARP4 expression positively correlates with the tumor suppressor LATS1, the direct target of miR-424-5p in gastric cancer [14]. Circ-ITCH, as a tumor suppressor in BC, could function as a ceRNA for miR-17 and miR-224 to promote p21 and PTEN expression [17]. However, miRNAs also function to positively regulate gene expression by targeting the 5’UTR [24]. In this study, miR-1178-3p was predicted to bind to the 5’UTR of G3BP2 by RegRNA2.0; A dual-luciferase reporter assay confirmed that miR-1178-3p could enhance G3BP2 expression via binding to its 5’UTR. Further study showed that overexpression of circFNDC3B significantly decreased the expression of G3BP2 in vitro and in vivo. G3BP2 has been reported to be overexpressed in BC cell lines [6] and to promote migration and invasion, as confirmed in our study. We speculated that regulatory networks of circFNDC3B/miR-1178-3p/G3BP2 could be involved in invasion and metastasis. First, miR-1178-3p was overexpressed in BC tissues and promoted the proliferation, migration and invasion of BC cells, which functionally exerted a negative correlation with circFNDC3B. Second, G3BP2 was validated as the direct target of miR-1178-3p. Third, overexpression of circFNDC3B partially abolished the effect of miR-1178-3p on G3BP2, implying a novel regulatory axis formed by circFNDC3B/miR-1178-3p/G3BP2 in BC.

Nonreceptor protein tyrosine kinases, such as FAK and SRC protooncogenes, play vital roles in integrin-associated signal transduction [32]. Aberrant activation of SRC/FAK signaling leads to enhanced migratory and invasive capabilities in many human tumors [33] and plays a pivotal role in tumor metastasis [8]. G3BP2 has been reported to activate the SRC/FAK signaling pathway. Therefore, during further studies, we observed that overexpression of circFNDC3B also inhibited the activity of SRC and FAK. Additionally, the effects of miR-1178-3p on SRC and FAK, which increased their activity, were abrogated upon overexpressing circFNDC3B simultaneously in BC cells compared with the control group. Furthermore, knockdown of G3BP2 could reverse sh-circFNDC3B-induced increase on p-SRC and p-FAK expression. These results indicate that circFNDC3B inactivates the SRC/FAK signaling pathway via miR-1178-3p/G3BP2.

## Conclusion

In summary, we identified an invasion-related circFNDC3B that was downregulated in human BC tissues and cells, and we found that decreased circFNDC3B expression was associated with poor prognosis of BC patients. Furthermore, we also demonstrated that overexpression of circFNDC3B could effectively inhibit cell proliferation and invasion in vitro and suppress tumor growth and lymphatic metastasis in vivo by targeting the miR-1178-3p/G3BP2/SRC/FAK axis, suggesting a key role for circFNDC3B in BC progression.

## Additional files

Additional file 1: **Table S1.** The sequences of primers, oligonucleotides and probes used in this study. (PDF 48 kb)

Additional file 2: **Figure S1.** circFNDC3B inhibits proliferation of BC cells. a and b qRT-PCR analysis for circFNDC3B and FNDC3B mRNA in UM-UC-3 cells treated with siRNAs or T24 cells transfected with circFNDC3B overexpression vector. c and d The effect of si-circFNDC3B on cell proliferation of UM-UC-3 cells was assessed by MTS and colony formation assays. e and f Assessment of cell proliferation of T24 cells transfected with circFNDC3B overexpression vector by MTS and colony formation assays. Data indicate means±SEM of three experiments. **P < 0.01 (Student’s *t*-test). **Figure S2.** circFNDC3B inhibits migration and invasion of BC cells. a and b The cell migratory and invasive capabilities were examined in UM-UC-3 cells treated with circFNDC3B siRNAs using wound healing assay, transwell migration and Matrigel invasion assays. c and d The cell migratory and invasive abilities were assessed after T24 cells were transfected with circFNDC3B overexpression vector. a and c, scale bar,200 μm; b and d, scale bar, 100 μm. Data indicate means±SEM of three experiments. *P < 0.05, **P < 0.01 (Student’s *t*-test). **Figure S3.** The identification and confirmation of circFNDC3B-related downstream molecules in BC cells. a The sequence alignment of miR-1178-3p with circFNDC3B. The mutant bases are depicted in red. b and c qRT-PCR analysis of 8 cancer-related genes after transfection with circFNDC3B siRNAs in T24 and UM-UC-3 cells. d The sequence alignment of miR-1178-3p with 3’UTR of p21 (predicted by Targetscan). e The sequence alignment of miR-1178-3p with 5’UTR of G3BP2. The mutant bases are depicted in red. Data indicate means±SEM of three experiments. *P < 0.05, **P < 0.01 (Student’s *t*-test). (PDF 13185 kb)

Additional file 3: The potential miRNAs targeted with circFNDC3B were predicted by CircInteractome. (XLSX 15 kb)

Additional file 4: The differentially changed genes were screened in both T24 and UM-UC-3 cells treated with circFNDC3B siRNA1 and siRNA2. (XLSX 19 kb)

## Abbreviations

BC: bladder cancer
CircRNAs: circular RNAs
FAK: focal adhesion kinase
G3BP: RasGAP SH3 domain-binding protein
LN: lymph node
qRT-PCR: quantitative real-time polymerase chain reaction
RasGAP: Ras-GTPase-activating protein
SRC: steroid receptor coactivator
