# Correction to: Invasion-related circular RNA circFNDC3B inhibits bladder cancer progression through the miR-1178-3p/G3BP2/SRC/FAK axis

**DOI:** 10.1186/s12943-020-01241-2

**Published:** 2020-08-10

**Authors:** Hongwei Liu, Junming Bi, Wei Dong, Meihua Yang, Juanyi Shi, Ning Jiang, Tianxin Lin, Jian Huang

**Affiliations:** Department of Urology and Guangdong Provincial Key Laboratory of Malignant Tumor Epigenetics and Gene Regulation, Sun Yat-sen Memorial Hospital, Sun Yat-sen University, 107th Yanjiangxi Road, Yuexiu District, Guangzhou, 510120 China

**Correction to: Mol Cancer 17, 161 (2018)**

**https://doi.org/10.1186/s12943-018-0908-8**

The authors regret that in the originally published article [1], Fig. 8e contains a duplicate image. They then proceed to re-check all original data, and found out that there was no duplication found in these files. The authors realized that the error happened during figure processing. All the original picture of Fig. 8e were saved in TIF format and were put in a folder for faster processing. During scale bar addition, they added a scale bar to the image of the control group using Image J software and saved it as JPG format picture in the same folder. Then, the four groups of pictures were combined and typeset by Adobe Illustrator CS 6 software. In the process of image processing, the control group picture without scale were dragged in by mistake, resulting in the repetition of the first picture and the third picture. This correction has not changed the interpretation or the original conclusions of this work. Hence, the authors truly apologize for the oversight on this matter to the editors, reviewers and readers for any confusion that has been caused by this unintentional error. The revised Fig. 8e is shown below.

**Fig. 8:**
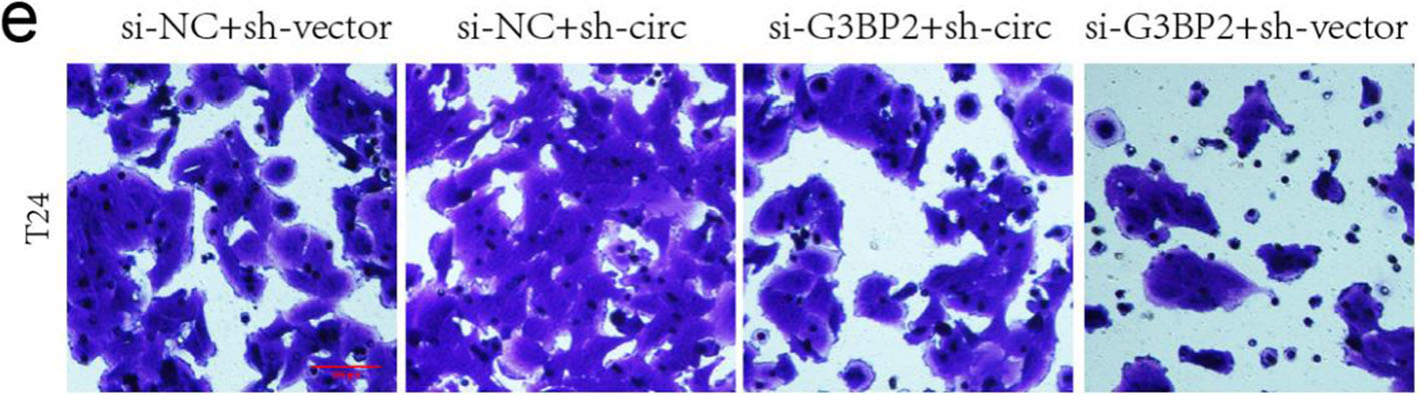
Knockdown of G3BP2 abolishes the oncogenic effects induced by downregulation of circFNDC3B. T24 or UMUC-3 cells were transfected with si-NC + sh-vector, si-NC + sh-circFNDC3B, si-G3BP2 + sh-circFNDC3B or si-G3BP2 + sh-vector. e The invasive capacity was evaluated by transwell Matrigel invasion assays. Scale bar, 200 μm
