# Supplementary material for: Invasion-related circular RNA circFNDC3B inhibits bladder cancer progression through the miR-1178-3p/G3BP2/SRC/FAK axis
Source: Mol Cancer. 2018 Nov 20;17:161. doi: 10.1186/s12943-018-0908-8 (PMC6245936; doi:10.1186/s12943-018-0908-8)
Supplement: Supplementary file 1 — Table S1. The sequences of primers, oligonucleotides and probes used in this study. (PDF 48 kb) [file 12943_2018_908_MOESM1_ESM.pdf]

Additional file 1: Table S1. The sequences of primers, oligonucleotides and probes used in this study

**Primers**	
circFNDC3B-F	CAAGAAGCAGCCCAAAGTCG
circFNDC3B-R	CATGGCTGAGGGGTAGCTTG
linear FNDC3B-F	ACTGAAAGACCGCCAGATCG
linear FNDC3B-R	TCTTGCTCGTCGCTCTGTTT
GAPDH-F	GGAGCGAGATCCCTCCAAAAT
GAPDH-R	GGCTGTTGTCATACTTCTCATGG
divergent GAPDH-F	GAAGGTGAAGGTCGAGTC
divergent GAPDH-R	GAAGATGGTGATGGGATTTC
CD82-F	CTGTTCCTGCGAAGTCAAGG
CD82-R	AGGATGATGCCCAGGTTCTC
P21-F	TGCCCAAGCTCTACCTTCC
P21-R	CAGGTCCACATGGTCTTCCT
NFKBIA-F	GTTGAAGTGTGGGGCTGATG
NFKBIA-R	GTCCTCTGTGAACTCCGTGA
NFKBIE-F	CATCTCACTCTCTGGACCTCC
NFKBIE-R	CCGAAGCAGCAATTCCATGA
G3BP2-F	AGAGGTGGTGGTGATGATCG
G3BP2-R	AGCCAAGTTTCTGTGCCATG
GANAB-F	GGATTGAGCGGGTGGTGATA
GANAB-R	TCTCAGGGTCATGCTGGAAG
SETD7-F	TGGGGAGATGACTGGAGAGA
SETD7-R	CCTCCCTTCTTCAGTGGACA
BICD2-F	GCTAAAGAAGGTGAGCGACG
BICD2-R	TAGTCCAGCATGACACGGTT
has-miR-587	TTTCCATAGGTGATGAGTCAC
has-miR-658	AGGGAAGTAGGTCCGTTGGT
has-miR-1270	CTGGAGATATGGAAGAGCTGTG
has-miR-1178-3p	TTGCTCACTGTTCTTCCCTAG
has-miR-1304-5p	TTTGAGGCTACAGTGAGATGTG
has-miR-510-5p	TACTCAGGAGAGTGGCAATCAC
has-miR-147b	GTGCGGAAATGCTTCTGCTA
has-miR-1298-5p	TCATTCGGCTGTCCAGATGTA
has-miR-644a	AGTGTGGCTTTCTTAGAGC
has-miR-1281	ATATCGCCTCCTCCTCTCCC
has-miR-578	CTTCTTGTGCTCTAGGATTGT
has-miR-625-5p	AGGGGGAAAGTTCTATAGTCC
has-miR-941	CGGCTGTGTGCACATGTGC
has-miR-1322	GATGATGCTGCTGATGCTG
has-miR-568	ATGTATAAATGTATACACAC
U6	CTCGCTTCGGCAGCACA

miR reverse	Sangon Biotech,China
**siRNAs**	
si-NC sense	UUCUCCGAACGUGUCACGUTT
si-NC antisense	ACGUGACACGUUCGGAGAATT
si-circFNDC3B-1 sense	CAGACUUGCAAGGUGAUUGTT
si-circFNDC3B-1 antisense	CAAUCACCUUGCAAGUCUGTT
si-circFNDC3B-2 sense	CUUGCAAGGUGAUUGAAGATT
si-circFNDC3B-2 antisense	UCUUCAAUCACCUUGCAAGTT
si-G3BP2-1 sense	GGAGGAGAAGAACUUAGAATT
si-G3BP2-1 antisense	UUCUAAGUUCUUCUCCUCCTT
**miR-1178-3p mimics and inhibitors**	
mimics NC sense	UUCUCCGAACGUGUCACGUTT
mimics NC antisense	ACGUGACACGUUCGGAGAATT
miR-1178-3p mimics sense	UUGCUCACUGUUCUUCCCUAG
miR-1178-3p mimics antisense	AGGGAAGAACAGUGAGCAAUU
inhibitor NC	CAGUACUUUUGUGUAGUACAA
miR-1178-3p inhibitor	CUAGGGAAGAACAGUGAGCAA
**FISH Probes**	
Cy3-circFNDC3	ACTATCTTCAATCACCTTGCAAGTCTGAAT
Cy3-U6	TTTGCGTGTCATCCTTGCG
Cy3-18S	CTTCCTTGGATGTGGTAGCCGTTTC
Cy5-hsa-miR-1178-3p	CTAGGGAAGAACAGTGAGCAA
**Biotinylated probes**	
Biotin-circFNDC3B	ACTATCTTCAATCACCTTGCAAGTCTGAAT
Biotin-miR-1178-3p	UUGCUCACUGUUCUUCCCUAG
Biotin-miR-1178-3p mutant	AACGAGUGUGUUCUUCCCUAG
