# Supplementary material for: Invasion-related circular RNA circFNDC3B inhibits bladder cancer progression through the miR-1178-3p/G3BP2/SRC/FAK axis
Source: Mol Cancer. 2018 Nov 20;17:161. doi: 10.1186/s12943-018-0908-8 (PMC6245936; doi:10.1186/s12943-018-0908-8)
Supplement: Supplementary file 2 — Figure S1. circFNDC3B inhibits proliferation of BC cells. a and b qRT-PCR analysis for circFNDC3B and FNDC3B mRNA in UM-UC-3 cells treated with siRNAs or T24 cells transfected with circFNDC3B overexpression vector. c and d The effect of si-circFNDC3B on cell proliferation of UM-UC-3 cells was assessed by MTS and colony formation assays. e and f Assessment of cell proliferation of T24 cells transfected with circFNDC3B overexpression vector by MTS and colony formation assays. Data indicate means±SEM of three experiments. **P < 0.01 (Student’s t-test). Figure S2. circFNDC3B inhibits migration and invasion of BC cells. a and b The cell migratory and invasive capabilities were examined in UM-UC-3 cells treated with circFNDC3B siRNAs using wound healing assay, transwell migration and Matrigel invasion assays. c and d The cell migratory and invasive abilities were assessed after T24 cells were transfected with circFNDC3B overexpression vector. a and c, scale bar,200 μm; b and d, scale bar, 100 μm. Data indicate means±SEM of three experiments. *P < 0.05, **P < 0.01 (Student’s t-test). Figure S3. The identification and confirmation of circFNDC3B-related downstream molecules in BC cells. a The sequence alignment of miR-1178-3p with circFNDC3B. The mutant bases are depicted in red. b and c qRT-PCR analysis of 8 cancer-related genes after transfection with circFNDC3B siRNAs in T24 and UM-UC-3 cells. d The sequence alignment of miR-1178-3p with 3’UTR of p21 (predicted by Targetscan). e The sequence alignment of miR-1178-3p with 5’UTR of G3BP2. The mutant bases are depicted in red. Data indicate means±SEM of three experiments. *P < 0.05, **P < 0.01 (Student’s t-test). (PDF 13185 kb) [file 12943_2018_908_MOESM2_ESM.pdf]

Figure S1

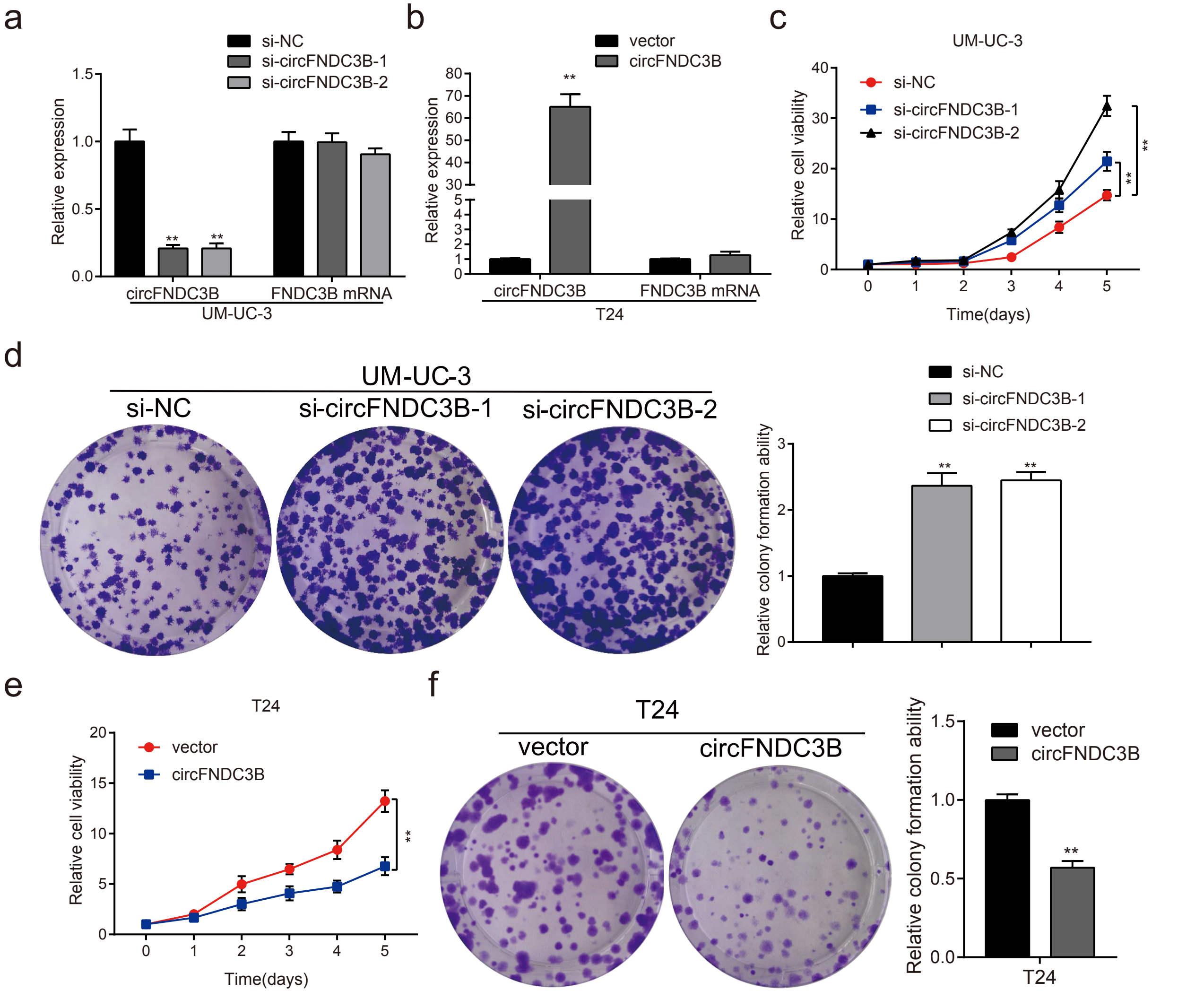

a
si-NC
si-circFNDC3B-1
si-circFNDC3B-2
Relative expression
circFNDC3B
FNDC3B mRNA
UM-UC-3
b
vector
circFNDC3B
Relative expression
circFNDC3B
FNDC3B mRNA
T24
c
UM-UC-3
si-NC
si-circFNDC3B-1
si-circFNDC3B-2
Relative cell viability
Time(days)
d
UM-UC-3
si-NC
si-circFNDC3B-1
si-circFNDC3B-2
Relative colony formation ability
e
T24
vector
circFNDC3B
Relative cell viability
Time(days)
f
T24
vector
circFNDC3B
Relative colony formation ability
T24

Figure S2

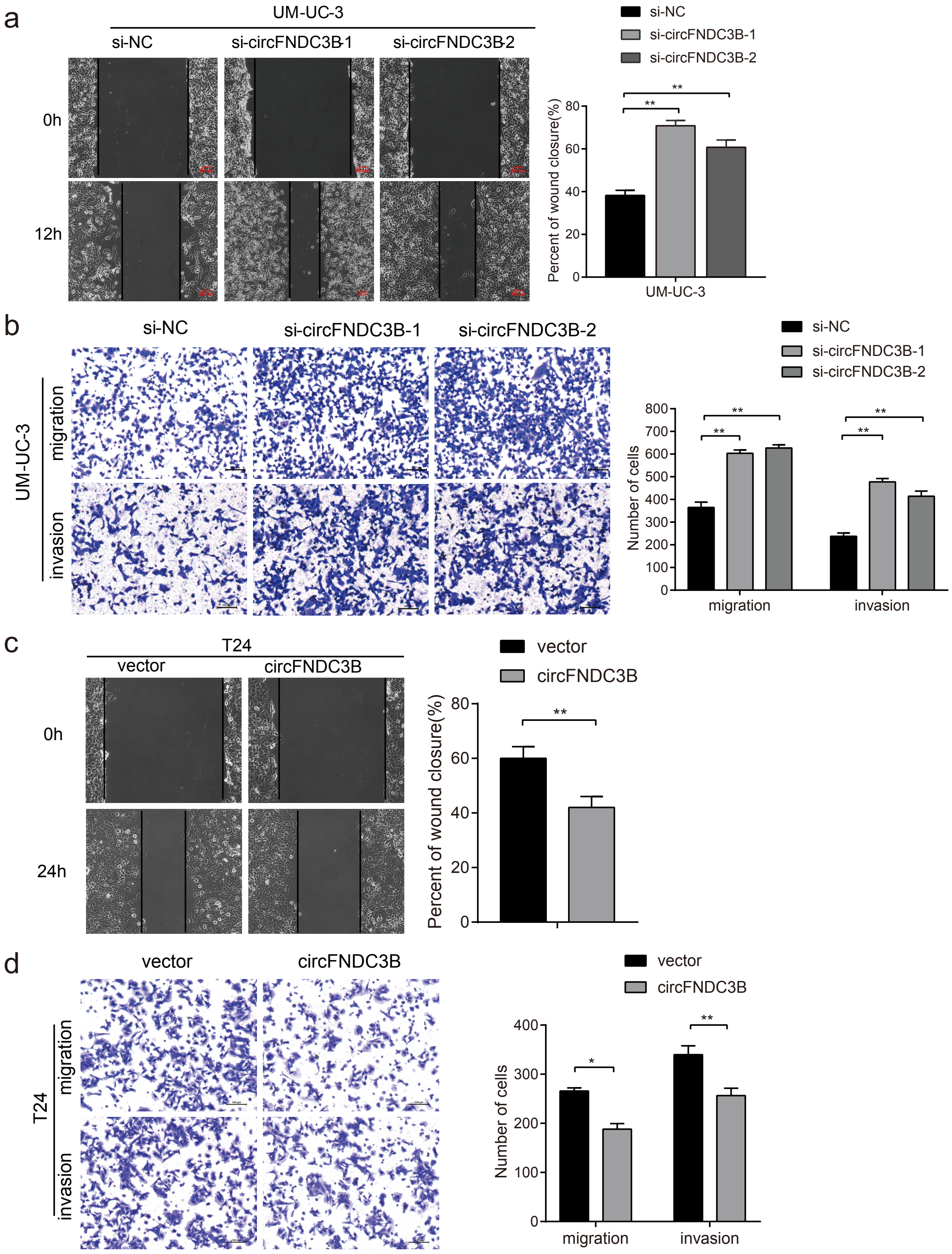

a
UM-UC-3
si-NC
si-circFNDC3B-1
si-circFNDC3B-2
0h
12h
si-NC
si-circFNDC3B-1
si-circFNDC3B-2
Percent of wound closure(%)
0
20
40
60
80
**
**
UM-UC-3
b
si-NC
si-circFNDC3B-1
si-circFNDC3B-2
UM-UC-3
migration
invasion
si-NC
si-circFNDC3B-1
si-circFNDC3B-2
Number of cells
0
100
200
300
400
500
600
700
800
**
**
**
**
migration
invasion
c
T24
vector
circFNDC3B
0h
24h
vector
circFNDC3B
Percent of wound closure(%)
0
20
40
60
80
**
d
vector
circFNDC3B
T24
migration
invasion
vector
circFNDC3B
Number of cells
0
100
200
300
400
*
**
migration
invasion

Figure S3

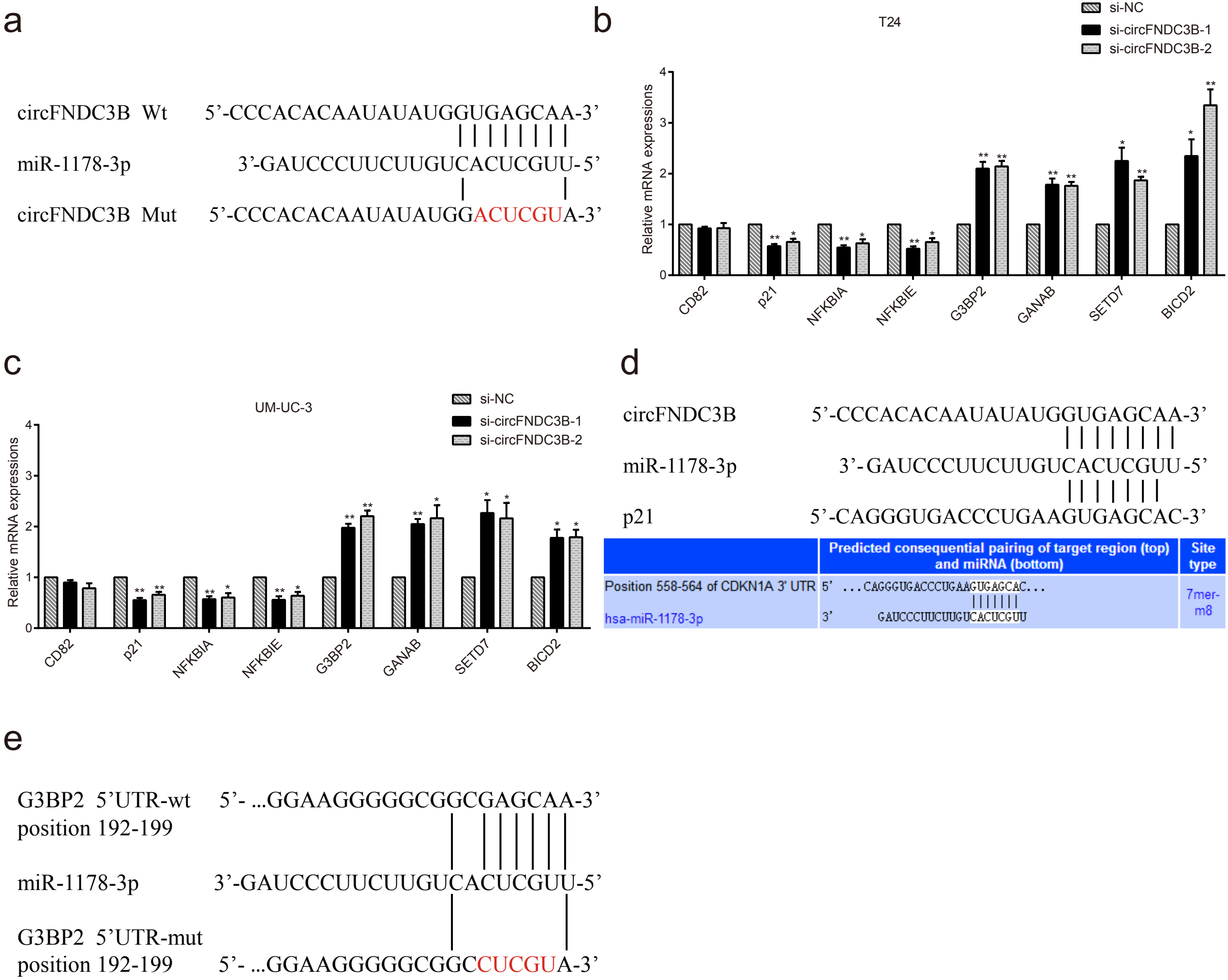
a
circFNDC3B Wt 5'-CCCACACAAUAUAUGGUGAGCAA-3'
miR-1178-3p 3'-GAUCCCUUCUUGUCACUCGUU-5'
circFNDC3B Mut 5'-CCCACACAAUAUAUGGACUCGUA-3'
b
T24
si-NC
si-circFNDC3B-1
si-circFNDC3B-2
Relative mRNA expressions
0
1
2
3
4
CD82
p21
NFKBIA
NFKBIE
G3BP2
GANAB
SETD7
BICD2
c
UM-UC-3
si-NC
si-circFNDC3B-1
si-circFNDC3B-2
Relative mRNA expressions
0
1
2
3
4
CD82
p21
NFKBIA
NFKBIE
G3BP2
GANAB
SETD7
BICD2
d
circFNDC3B 5'-CCCACACAAUAUAUGGUGAGCAA-3'
miR-1178-3p 3'-GAUCCCUUCUUGUCACUCGUU-5'
p21 5'-CAGGGUGACCCUGAAGUGAGCAC-3'
Predicted consequential pairing of target region (top) and miRNA (bottom)
Site type
Position 558-564 of CDKN1A 3' UTR
5' ...CAGGGUGACCCUGAAGUGAGCAC...
hsa-miR-1178-3p
3' GAUCCCUUCUUGUCACUCGUU
7mer-m8
e
G3BP2 5'UTR-wt position 192-199 5'- ...GGAAGGGGGCGGCGAGCAA-3'
miR-1178-3p 3'-GAUCCCUUCUUGUCACUCGUU-5'
G3BP2 5'UTR-mut position 192-199 5'- ...GGAAGGGGGCGGCCUCGUA-3'
